# L-Asparaginase Activity in Cell Lysates and Culture Media of Halophilic Bacterial Isolates

**Published:** 2016

**Authors:** Mahmood Barati, Mohammad Ali Faramarzi, Nastaran Nafissi-Varcheh, Mohammad Reza Khoshayand, Mohammad Hassan Houshdar Tehrani, Hossein Vahidi, Sina Adrangi

**Affiliations:** a *Department of Pharmaceutical Biotechnology, School of Pharmacy, Shahid Beheshti University of Medical Sciences, Tehran, Iran. *; b *Department of Pharmaceutical Biotechnology, Faculty of Pharmacy and Biotechnology Research Center, Tehran University of Medical Sciences, P.O. Box 14155-6451, Tehran, 14176, Iran. *; c *Department of Drug and Food Control, Faculty of Pharmacy, Tehran University of Medical Sciences, Tehran, Iran. *; d *Department of Medicinal Chemistry, School of Pharmacy, Shahid Beheshti University of Medical Sciences, Tehran, Iran.*

**Keywords:** Screening, Halophile, L-asparaginase, *Halomonas*, *Aidingimonas*

## Abstract

The objective of this study was to isolate halophilic bacteria with the ability to produce intracellular or extracellular L-asparaginase. A total number of 120 halophilic bacteria were isolated from 17 different saline habitats of Iran including salt lakes, wetlands, brine springs and deserts. Among these, 68 were able to grow in the presence of 1.5 M NaCl and 52 demonstrated the ability to grow in the selection medium containing 3.5 M NaCl. None of the isolates appeared to produce appreciable amounts of extracellular L-asparaginase. Among the isolates that produced intracellular L-asparaginase, 5 moderate and 1 extreme halophiles were selected for further study based on their observed activity level. The moderately halophilic isolates were shown to belong to the genus *Halomonas* while the extreme halophile was identified as a member of the genus *Aidingimonas*.

## Introduction

L-Asparaginase (EC 3.5.1.1) catalyzes the hydrolysis of asparagine to aspartate and ammonia and is found in a wide range of organisms including mammals, birds, plants, and microorganisms. Owing to their ability to specifically remove asparagine from complex systems, L-Asparaginases have found a number of applications in medicine and food industries (1-4). For example, L-asparaginases derived from *Escherichia coli* and *Erwinia chrysanthemi* are an essential part of treatment protocols for acute lymphoblastic leukemia (4-7). They exert their effect by depleting the extracellular L-asparagine pool required for the growth and proliferation of cancer cells. Unlike tumor cells, normal cells do not depend on extracellular L-asparagine. L-Asparaginases are also used to prevent the formation of acrylamide in heat-treated foods (8-10). During high-temperature cooking, asparagine and reducing sugars may react via the Maillard reaction to produce acrylamide (11). Although the significance of such low levels of acrylamide is not yet completely understood, the food industry has voluntarily taken some steps to further reduce acrylamide levels in final products due to concerns regarding its carcinogenicity (12). A major strategy toward this end is to pretreat raw material with L-asparaginase to reduce asparagine levels and a few commercial enzyme preparations for this purpose are available (13, 14). However, such industrial-scale enzymatic processes are often performed under harsh conditions such as high temperature or salinity that may deactivate enzymes obtained from ordinary microorganisms. Enzymes produced by halophilic or halotolerant microorganisms, on the other hand, tend to show higher tolerance to extreme conditions such as high salt concentrations, high temperatures, non-physiological pH values and the presence of organic solvents (15). This study was conducted in an attempt to identify halophilic\halotolerant microorganisms capable of producing L-asparaginases with potential industrial applications.

**Table 1:** Moderate and extreme halophiles isolated in this study and localization of their L-asparaginase activity.

**Type of halophile**	**Number of isolates**					
	**With L-asparaginase activity**	**With intracellular activity only**	**With extracellular activity only**	**With both intra and extracellular activity**	**Without L-asparaginase activity**	**Total**
Moderate	21	13	0	8	47	68
Extreme	17	12	0	5	35	52
Total	38	25	0	13	82	120

**Table 2:** Characteristics of isolates showing the highest level of L-asparaginase activity among all isolates investigated in this study

**Isolate**	**Growth medium NaCl concentration (M)**	**Intracellular L-asparaginase activity (U/mL)**	**extracellular L-asparaginase activity (U/mL)**	**Identification results**
H2	1.5	1.2	0.2	*Halomonas*
H3	1.5	1.6	0.2	*Halomonas*
H23	1.5	1.4	0.3	*Halomonas*
H27	1.5	1.9	0.2	*Halomonas*
H28	1.5	2.4	0.2	*Halomonas*
H33	3.5	1.3	0.1	*Aidingimonas*

## Experimental

*Screening and isolation of halophilic microorganisms exhibiting* *L-* *asparaginase activity*

Soil and water samples were collected in sterile containers. In order to isolate moderate and extreme halophilic bacteria from soil samples, samples were suspended in phosphate buffer (pH 7.0) supplemented with 1.5 M and 3.5 M NaCl, respectively, and briefly incubated on an orbital shaker at room temperature. Samples were then centrifuged to separate solid particles and the supernatant was used to streak Luria-Bertani agar plates supplemented with 1.5 M or 3.5 M NaCl. To ensure clone purity, all isolates were subjected to successive rounds of streak plating. Water samples were processed in a similar way except that they were first filtered to separate solid particles and then directly transferred to screening plates.

L-Asparaginase-producing microorganisms were screened using the plate assay method (16). Briefly, halophilic isolates were transferred to screening plates containing a modified M9 agar medium (pH 7.0) supplemented with L-asparagine (10 g/L), either 1.5 M or 3.5 M NaCl and the pH indicator phenol red (0.009%). Plates were then incubated at 37 °C for at least 48 h. L-Asparaginase activity results in an increase in medium pH that can be detected by the formation of pink zones around the colonies.

*Cell fractionation*

Bacterial cells were inoculated into 20-mL volumes of the modified M9 medium described above (minus phenol red and agar) and incubated at 37 °C and 180 rpm for 24 h. Cultures were then centrifuged at 10000*g* for 5 min at 4 °C. The culture supernatants were used to determine the L-asparaginase activity in the extracellular compartment. The collected cells were washed twice in 50 mM Tris-HCl buffer (pH 8.5) and disrupted using an ultrasonic homogenizer. The lysates were clarified by centrifugation at 13000*g* for 10 min at 4 °C. The supernatants were used to measure intracellular enzyme activity.

*L-* *Asparaginase assay*

L-Asparaginase activity was measured by the Nessler’s reagent method (17). In brief, 100 μL of enzyme preparation was mixed with 400 μL of distilled water, 500 μL of 50 mM Tris-HCl buffer (pH 8.6) supplemented with NaCl (final concentration of 0-3.5 M) and 100 μL of 94.5 mM asparagine solution and incubated at 37 °C for 30 min. The enzyme reaction was stopped by adding 50 μL of trichloroacetic acid (1.5 M). An aliquot of 100 μL of the above mixture was added to 2150 μL of distilled water and 250 μL of Nessler’s reagent (Sigma-Aldrich). The absorbance was measured at 436 nm. One enzyme unit was defined as the amount of enzyme that produces 1 μmol of ammonia in 1 min under assay conditions.

*Identification*

In order to extract genomic DNA, 2-3 fresh colonies of each isolate grown on modified M9 plates were mixed, suspended in distilled water and incubated in a boiling water bath for 10 min. The lysate was centrifuged at 12000*g* for 10 min and the supernatant was used as template. A region of approximately 1500 pb of the 16S rDNA gene was amplified using primers 27F (5’-AGAGTTTGATCCTGGCTCAG-3’) and 1492R (5’-TACGGCTACCTTGTTACGACTT-3’) (18, 19). The PCR reactions were carried out in a thermocycler (peqSTAR, PEQLAB Biotechnologie, Germany) with the following conditions: initial denaturation 95°C for 300 s, followed by 30 cycles of 95 °C for 60 s, 60 °C for 60 s and 72 °C for 90 s and final extension 72 °C for 600 s. The amplified region was sequenced using primers 27F, 518F (5’-CAGCAGCCGCGGTAATA-3’) and 1492R (18, 19). Isolates were identified by comparing the obtained 16S rDNA sequences with the NCBI 16S Ribosomal RNA Sequences database.

## Results and discussion

*Screening and isolation*

Several water and soil samples were collected from 17 hypersaline environments in Iran including 9 salt lakes (Aran va Bidgol, Hoze soltan, Damghan, Khur, Selkenoon, Golestan, Shoor Mast, Shourabil and Urmia), 4 wetlands (Ajigol, Alagol, Almagol and Gomishan), 2 brine springs (Abhar and Kal Shoor) and 2 deserts (Maranjab and Mesr) between 2010 and 2011. A total number of 120 halophilic bacterial isolates were retrieved from these samples using the screening procedure described in the Experimental section. Although the definition of moderate and extreme halophiles remains somewhat arbitrary, microorganisms that grow best at 0.8 M to 3.4 M are usually considered moderate halophiles while those that require more than 3.4 M salinity are classified as extreme halophiles (20). Using this classification scheme as a benchmark, we investigated the ability of isolates to grow in the presence of 1.5 M and 3.5 M NaCl. Among the 120 isolates, 68 were able to grow in the presence of 1.5 M NaCl and 52 demonstrated the ability to grow in the selection medium containing 3.5 M NaCl. These findings are summarized in Table 1. As described in the following sections, both groups are considered potential sources for industrial enzymes.

*L-* *Asparaginase activity*

Approximately one-third of both moderate and extreme halophilic isolates showed asparaginase activity on agar plates (Table 1). Thus, a total number of 38 halophilic isolates with L-asparaginase activity were obtained. Since the agar plate method may produce false positive results, the presence of L-asparaginase activity in these isolates was also investigated and confirmed using the Nessler assay.

Since bacteria have been reported to produce both intracellular and extracellular L-asparaginases, all isolates were screened for the presence of both intra and extracellular activity. Most isolates showed only intracellular L-asparaginase activity. Only 13 isolates tested positive for extracellular L-asparaginase activity, all of which also demonstrated the ability to produce intracellular L-asparaginase. In all cases, the extracellular activity was very low. Although these observations may have resulted from the suboptimal assay conditions, they may also suggest that the extracellular activity actually results from leaked or released periplasmic or membrane-bound enzymes rather than actively secreted ones. It may at first seem counterintuitive to base a screening program for halotolerant L-asparaginases on intracellular enzymes; however, as described in the next section, even intracellular enzymes from moderate halophiles may demonstrate very useful properties. As seen in Table 1. the relative abundance of the strains with extracellular L-asparaginase activity does not appear to be de related to their salt tolerance.

*Identification*

Most of the isolates demonstrated very low levels of L-asparaginase activity. As the objective of this study was to identify halophilic bacterial strains with potential industrial applications, only those producing high enzyme levels were selected for further study. Since a relatively large difference in L-asparaginase activity between isolates producing more than 1 U/mL and those producing lower levels was observed, this level was chosen as a threshold. None of the halophilic isolates with extracellular L-asparaginase activity showed satisfactory activity levels. This is rather unfortunate in that it is actually this group of enzymes that are exposed to hypersaline conditions (21). In addition, the purification of extracellular enzymes is usually less challenging than their intracellular counterparts. However, intracellular enzymes from halophiles may also show higher tolerance to unusual conditions compared to those obtained from other microorganisms depending on the osmoregulation mechanisms used by their hosts. Halophilic microorganisms use two different strategies to maintain proper cytoplasmic osmotic pressure when exposed to high salinity (22, 23). The first mechanism is known as the salt-in strategy and involves the accumulation of high concentrations of KCl in the cytoplasm that, depending on growth conditions, may exceed 2 M. This strategy is not very common and is only used by a few species from the orders *Halobacteriales* and *Haloanaerobiales* (22). In this group of halophilic microorganisms, the intracellular enzymes have adapted to high salt concentrations and show remarkable halotolerance. A number of such enzymes have been characterized so far (24, 25). The second mechanism used by halophiles to cope with their unusual environment, usually referred to as the compatible-solute strategy, is to synthesize high levels of specialized organic solute known as osmolytes. These are neutral and highly soluble low molecular mass molecules that not only do not interfere with enzymatic activity, but provide some protective effect against denaturing conditions (23, 26). Although from a bioenergetics view point this strategy is less favorable than the first one, it has been more widely adopted by halophilic microorganisms since, at least theoretically, it does not require any adaptation of the intracellular enzymes (22). In practice, however, some intracellular enzymes from these organisms also appear to show significant salt tolerance (27). This is probably due to the fact that even halophilic species that predominantly use the compatible-solute strategy may accumulate high concentration of potassium ions under certain conditions (28). In this study, among isolates with intracellular L-asparaginase activity, 6 isolates showed activity levels higher than the selected threshold of 1 U/mL. As indicated in Table 2, 5 isolates are moderate halophiles (H2, H3, H23, H27 and H28) while the last one is an extreme halophile (H33). These isolates were identified through 16S rDNA sequencing. Although 16S rDNA sequencing is not as accurate as robust identification methods such as DNA-DNA hybridization, it has widely been used in the identification of unknown bacteria isolated from clinical or environmental samples mostly due to its ease of use, affordability and overall reliability (29). It is of interest to note that despite the fact that they were isolated from geographically distant locations, 5 out of 6 isolate identified in this study turned out to belong to the same genus (i.e., *Halomonas*). This may have resulted from the enrichment effect of the selection medium used in this study. The M9 medium used for the isolation of moderate halophiles was supplemented with 1.5 M (8.7%) NaCl. This is very close to the optimal concentration of 8% recommended for *Halomonas* cultivation (20). Other minor constituents may also have contributed to this effect. It is probably impossible to avoid this phenomenon completely as no medium can support the growth of all halophilic species due to their different requirements (20).

*Halomonas* species are moderate halophiles that use the compatible-solute strategy for osmotic adaptation (26). However, they have also been shown to use K^+^-dependent mechanisms (28). In fact, reports about the presence of halotolerant intracellular enzymes in *Halomonas* species have already been published (30). The five *Halomonas* strains isolated in the present study may thus be of potential value.

H33, on the other hand, most likely belongs to the genus *Aidingimonas*. *Aidingimonas* has been described as a moderately halophilic bacterium (31). Yet, in the same study, it has been reported to tolerate NaCl concentrations as high as 4 M which is in accordance with our observations. Unlike *Halomonas*, *Aidingimonas* is not extensively studied or characterized. However, *Aidingimonas *probably also uses the compatible-solute strategy as it is a member of the family Halomonadaceae (32). Although apparently no salt tolerant enzymes have been identified in this genus to date, this probably stems from the fact that very few studies about the enzymatic capabilities of this genus have been conducted. As any other bacterium with the ability to thrive in hypersaline environments, *Aidingimonas* may well contain a number of intracellular halotolerant enzymes.

The halophilic *Halomonas* and *Aidingimonas* strains isolated in this study provide a potential source of L-asparaginase for industrial applications. Studies are underway in our laboratory to optimize enzyme production by these isolates and to purify and characterize the enzymes responsible for the observed L-asparaginase activity. Of paramount importance, is the salt tolerance of these enzymes that, if observed, would also help better understand the enzymatic properties of less studied isolates such as *Aidingimonas*.
